# Standup comedy principles and the personal monologue to explore interpersonal bias: experiential learning in a health disparities course

**DOI:** 10.1186/s12909-022-03139-7

**Published:** 2022-02-05

**Authors:** Marshall H. Chin, Mona M. Aburmishan, Mengqi Zhu

**Affiliations:** 1 Section of General Internal Medicine, Department of Medicine, University of Chicago, 5841 South Maryland Avenue, MC2007, Chicago, IL 60637 USA; 2 MacLean Center for Clinical Medical Ethics, Chicago, IL USA; 3 Bucksbaum Institute for Clinical Excellence, University of Chicago, Chicago, IL USA; 4 Mona Comedy Inc., Chicago, IL USA; 5 Northeastern Illinois University, Chicago, IL USA

**Keywords:** Equity, Disparities, Racism, Comedy, Education

## Abstract

**Background:**

Interpersonal biases between clinicians and patients contribute to disparities in health care and outcomes by race, ethnicity, and socioeconomic status. We used standup comedy principles and exercises to help medical students recognize how others perceive them and how they perceive others, and engage in difficult discussions around implicit biases and interpersonal racism.

**Methods:**

90 min Zoom workshop with 40 first-year medical students in urban medical school. Intervention consisted of three exercises: Naming icebreaker, Rant and Rave (communicate strong perspective clearly), and Personal Monologue about how others perceive you and how you perceive yourself. Discussion debriefed the personal monologue exercise. Likert scale questions on post-session survey evaluated workshop overall, whether workshop increased skills, and safety of learning environment. Open-ended questions included what trainees liked about the module, what could be improved, and what impact the module had on them?

**Results:**

Seventeen (42.5%) students responded to survey. Six respondents identified as white, 4 as Asian, 1 as Black, 1 as multiracial, and 5 did not identify. Seventy-six percent rated the module as “very good” or “excellent”, and 94% would recommend the module to others. Most respondents reported the workshop helped them become better listeners (75%) and more observant (82%). Eighty-three percent reported the training could help them take better care of patients with lived experiences different than their own. All respondents believed the learning environment was safe, and 94% reported that instructors created an atmosphere in which they could take risks. Thirty-six percent felt stressed. Students reported the workshop helped them recognize their own identities, others’ perceptions, and bidirectional biases, and inspired them to strive for more accurate, authentic interactions with patients.

**Conclusions:**

Standup comedy principles show promise for engaging students in meaningful, safe discussions about perceptions and interpersonal biases rooted in their own personal experiences and those of their classmates.

**Supplementary Information:**

The online version contains supplementary material available at 10.1186/s12909-022-03139-7.

## Background

National awareness of systemic racism in the United States has greatly increased because of the publicity around long-standing police brutality against racial/ethnic minoritized populations and inequities in COVID-19 outcomes [1, 2]. Simultaneously toxic national discord has escalated; divided populations believe diametrically opposed “alternative facts” and clash with one another. To advance health equity, it is critical to engage in free, frank, and fearless discussions about perceptions, biases, and systemic racism [3, 4]. Ideal discussions would be personal, experiential, honest, and safe, and uncover hidden agendas that drive racially biased behavior [5]. Yet, the acrimonious environment makes it more challenging than ever to hold such dialogues [6].

Standup comedy uses rehearsed scripted and improvisational storytelling [7]. To be successful, standup comedians must have self-insight and recognize how the audience perceives them. We believe that exercises and principles used to train modern American standup comedians could engage health professional trainees to collectively discover perceptions and belief systems, and explore the world’s truths and absurdities [8]. Standup comedy exercises around perceptions could engage trainees in difficult discussions around interpersonal biases and improve their skills communicating and developing relationships with diverse patients [9, 10].

One of us (MA) is a Muslim American/British Palestinian professional standup comedian and comedy instructor who performs throughout the North America, United Kingdom, European Union, South Africa, and Middle East. Another (MC) is a Chinese American physician, health equity researcher, and amateur improvisational and standup comedian who performs in Chicago. We recognized that effective standup comedians bond with an audience and create a safe space for dialogue. Effective standup comedians recognize how they perceive their audiences as well as how audiences perceive them, and adapt their communication accordingly. Failure to size up an audience correctly will quickly lead to the comic failing in their act, with little laughter, outright retraction of the viewers’ attention, or people walking out of the venue. Such failure is analogous to the clinician who misunderstands or inadvertently disrespects a patient, causing the patient to disengage, ignore the clinician’s recommendations, or even seek a new provider.

The successful comedian is authentic and engenders trust and connection through their truth-telling, relatability, and vulnerability. Standup comedy teaches one objective is paramount: leave the audience better than you found it. Therefore, standup comedians cast a mirror to the inherent bidirectional biases laden in most environments between comedian and audience, analogous to the powerful spoken and unspoken perceptions between clinician and patient [11]. They learn how to create a safe space among strangers. From our academic work improving shared decision making between clinicians and marginalized, minoritized populations [11–14], and our experience performing standup comedy to diverse audiences, we learned how understanding these bidirectional biases between people is crucial for effective communication and relationship-building.

Thus, standup comedy training exercises around perceptions could be an innovative way to improve clinicians’ abilities to recognize how diverse patients perceive them and how they perceive their patients. Such training could provide an entry into difficult discussions around implicit biases and interpersonal racism [15, 16]. Different art forms such as theater, music, visual arts, and improvisational comedy have been used in health professions education [17], and standup techniques have been proposed to improve the effectiveness of nursing education presentations [18]. However, to our knowledge, standup comedy training exercises and principles have not previously been used to teach about advancing health equity. In an exploratory pilot, we tested standup training techniques for gaining self-insight and exploring interpersonal biases in 90 min Zoom workshops for first-year medical students. We hypothesized that students would perceive the workshop: 1) provided them skills to care for diverse patients, 2) encouraged meaningful discussions about systemic inequities, and 3) created a safe learning environment.

## Methods

### Workshop

As part of the University of Chicago Pritzker School of Medicine required first-year medical student course “Health Disparities: Equity and Advocacy” [19], 2 sequential 90-min Zoom sessions were held September 15, 2020 in which every student was randomly assigned to 2 of the following 4 art forms: standup comedy, improvisational comedy, graphic medicine, and Theater of the Oppressed. Our paper analyzes the 40 students who were assigned to one of the standup sessions.

Prior to the session, the students were assigned two articles that describe spoken and unspoken perceptions and biases inherent in clinician-patient encounters and how improvisational and standup comedy could help clinicians better care for diverse patients [11, 20]. They were told at the beginning of the workshop: “You may be wondering why you are in a standup comedy session in medical school at the University of Chicago. Don’t worry - we aren’t trying to get you booked at the local comedy club. We’re excited to share with you fun, innovative and empowering techniques and principles used in professional standup comedy that will help you “Read the Room” and “Read Yourselves,” critical skills for caring for diverse patients effectively.”

At the beginning of each session, we shared upfront ground rules: “First, you don’t need to be funny and today’s session is not really about comedy. It’s about gaining more insight into yourself, others, and your interaction with others. Second, we don’t want anyone to feel uncomfortable. So, if anyone would prefer not doing an exercise and rather observe and perhaps comment and contribute that way – that’s OK. Third, everyone should feel comfortable disclosing as much or as little about themselves as they feel comfortable. A good standup is authentic and vulnerable, and it can take a while for the comic to feel comfortable in their skin. So, please share whatever you feel comfortable sharing. We want this to be a safe space where we assume good intentions by all and we are aiming for constructive discussions to help us all grow.”

The workshop consisted of three exercises (Table 1): 1) Naming Icebreaker; 2) Rant and Rave, an exercise that trains us to take a strong perspective, think creatively, and communicate our thoughts. Rant and Rave was designed to be a fun warmup for the key exercise; 3) Personal Monologue that would require authenticity, vulnerability, and genuine self-reflection to be most effective. After about six volunteers presented their monologues, we then engaged in a facilitated discussion centering around: 1) Authenticity – knowing yourself; 2) Awareness of disconnect between how you perceive yourself and how others perceive you; 3) Implications for developing effective, respectful clinician-patient communication and relationships. We chose these core exercises and facilitation questions after discussing and piloting more extensive options. These exercises and discussion questions do not require extensive standup comedy training to implement. A brief demonstration of a personal monologue is available on the Bucksbaum Institute YouTube channel [21].

**Table 1:** Workshop Exercises and Discussion Questions

**Naming Icebreaker**	
1) State your name	
2) Share a very brief story about your name or else a fun fact about yourself	
**Rant and Rave**	
Your classmates are going to give you a suggestion for an item you might find in a house – like an alarm clock or a spoon. Your classmates will also tell you whether you love this item or hate it. Then you will have 1 min to rant about why you hate it or rave about why you love it.	
**Personal Monologue**	
We’ll be exploring our own personal monologue by first journaling for 5 min, then volunteers will do 90 s maximum monologues.	
Please choose one of the following 3 options to journal and prepare a monologue about:	
Option 1–1. How do others perceive me when they first meet me? 2. What do they get right? 3. What do they get wrong?	
Option 2–1. What are 3 adjectives people use to describe me when they first meet me? 2. What do they get right? 3. What do they get wrong?	
Option 3 - Please tell a story about a time when there was a mismatch between how someone perceived you and how you really are.	
**Discussion Questions After Personal Monologue Exercise**	
How did you find this journaling and monologue exercise?	
As you think about your own story and the monologues of your classmates, what did you notice?	
[Eventually discussion will get to the mismatch between how others perceive you and how you perceive yourself]	
Why does this disconnect matter?	
What does this disconnect mean for how we communicate with people?	
[Realization that perception of self has been shaped by American stereotypes]	

One session lost time because of technical difficulties assigning students to the standup videoconference room, and therefore the Rant and Rave exercise was removed from that session.

### Evaluation

The last 5 min of each 90 min session, the students were asked to complete a brief online questionnaire about that specific session (Appendix). The survey included 5-point Likert scale questions informed by Watson’s survey to evaluate her improvisational comedy workshop for medical students as well as our own questions tailored for the health disparities context [22]. Questions covered skills, systemic inequities, learning environment, and overall rating. The survey also asked several open-ended questions including: What you liked about the module? What could be improved? What impact did the module have on you? The same survey was used for the standup comedy, improvisational comedy, graphic medicine, and Theater of the Oppressed workshops. Three email reminders were sent to complete the survey.

### Quantitative and qualitative data analysis

We tabulated descriptive statistics and used the nonparametric Wilcoxon test to compare numerical results across categories of student gender and race/ethnicity, and first versus second administration of the workshop [23]. One author (MC) analyzed open-ended comments to identify initial themes, and the other authors reviewed raw data and themes to reach consensus [24].

## Results

Seventeen (42.5%) of the 40 students responded to the survey. Ratings between Session 1 (without Rant and Rave; *n* = 11) and Session 2 (with Rant and Rave; *n* = 6) were similar and trending higher with Session 2.

### Student demographics

Nine (53%) respondents identified as female, 5 (29%) as male, and 3 (18%) did not respond. Six (35%) respondents identified as white, 4 (24%) as Asian, 1 (6%) as Black, 1 (6%) as multiracial, and 5 (29%) did not identify. In the entire medical school class (*n* = 90), 47 (52%) identified as female, 25 (28%) as white, 33 (37%) as Asian, 15 (17%) as Black, 10 (11%) as Latino, 4 (4%) as other, and 3 (3%) did not identify.

### Quantitative questions

**Fig. 1:**
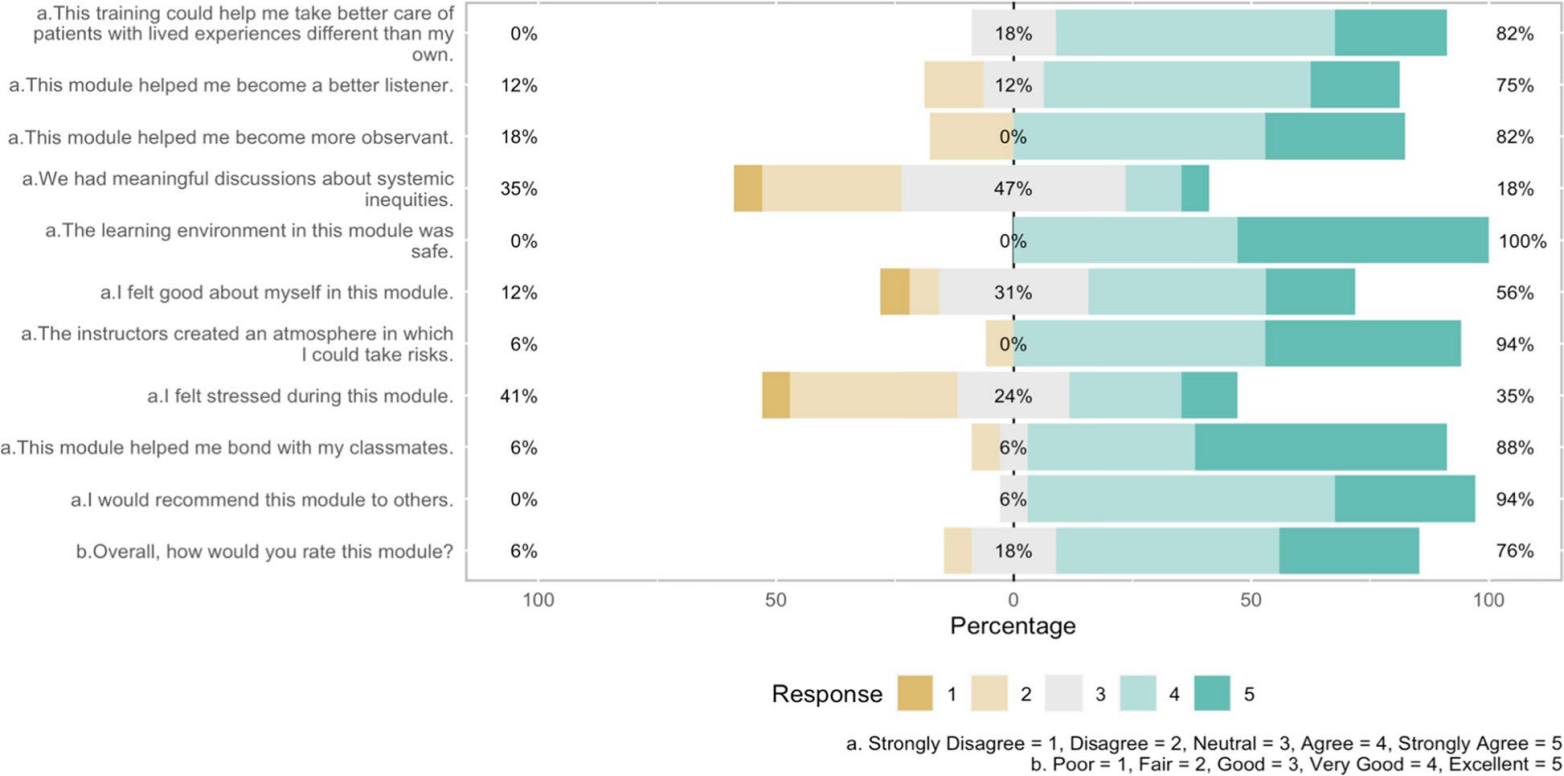
Student Perceptions of Standup Workshop: Bar Plot of Quantitative Survey Results (*n* = 17 respondents)

Seventy-six percent of the 17 respondents rated the module as “very good” or “excellent”, and 94% would recommend the module to others (Table 2, Fig. 1). Most respondents reported that the workshop helped them become better listeners (75%) and more observant (82%), and that it helped them bond with their classmates (88%). Eighty-three percent reported that the training could help them take better care of patients with lived experiences different than their own. Eighteen percent of the respondents agreed “We had meaningful discussions about systemic inequities.”

**Table 2:** Student Perceptions of Standup Workshop: Quantitative Survey Results (n = 17 respondents)

Strongly				Strongly		
Disagree	Disagree	Neutral	Agree	Agree	Mean^a^	SD
**Skills**						
This training could help me take better care of patients with lived experiences different than my own.						
0	0	18	59	24	4.06	0.66
This module helped me become a better listener.						
0	13	13	56	19	3.81	0.91
This module helped me become more observant.						
0	18	0	53	29	3.94	1.03
**Systemic Inequities**						
We had meaningful discussions about systemic inequities.						
6	29	47	12	6	2.82	0.95
**Learning Environment**						
The learning environment in this module was safe.						
0	0	0	47	53	4.53	0.51
I felt good about myself in this module.						
6	6	31	38	19	3.56	1.09
I felt stressed during this module.						
6	35	24	24	12	3.00	1.17
This module helped me bond with my classmates.						
0	6	6	35	53	4.35	0.86
The instructors created an atmosphere in which I could take risks.						
0	6	0	53	41	4.29	0.77
**Overall**						
I would recommend this module to others.						
0	0	6	65	29	4.24	0.56
Poor	Fair	Good	Very Good	Excellent	Mean^b^	SD
Overall, how would you rate this module?						
0	6	18	47	29	4.00	0.87

^a^ Strongly Disagree = 1, Disagree = 2, Neutral = 3, Agree = 4, Strongly Agree = 5

^b^ Poor = 1, Fair = 2, Good = 3, Very Good = 4; Excellent = 5

All 17 respondents believed the learning environment was safe and 94% reported that instructors had created an atmosphere in which they could take risks. Thirty-six percent felt stressed during the modules.

### Open-ended qualitative questions

We identified three major themes from the qualitative data: 1) Self-identity, misperceptions, and the danger of biases; 2) Space for safe, brave, fun discussions; 3) Value of standup and the non-medical context.

### Self-identity

Self-reflection led to insight.

*This module encouraged self-reflection & vulnerability in a way that felt both safe & fun. It was truly engaging, and I felt that it lent itself especially well to self-insight (Student 74).*

Deeper understanding of self came from both inwards thought as well as recognition of how others perceive and interact with them.

*I greatly appreciated having the opportunity to think about how I perceive myself and how others perceive me in a structured context. I feel that this is very important to breaking down my role in the clinical encounter (Student 77).*

*It helped me consider what others thought of me and how I present myself to others (Student 72).*

*It made me feel more curious about myself as I relate to others and how I can create consonance between my vision of myself/my values and my expression of that self/those values in the real world (Student 74).*

Some students recognized how white social privilege and power gave them the luxury of lack of self-awareness.

*This was a helpful reminder that I am not often forced to think about how people perceive me because I am in the dominant racial group and thus am afforded the privilege of un-self-consciousness (Student 74).*

### Misperceptions

Some students realized they lacked insight into how others viewed them.

*I think it forced me to confront some of my own blindness regarding others’ perceptions of myself (Student 67).*

### Dangers of biases

Students recognized how biases could lead to misunderstandings and harm people.

*I think that this made me realize that other people also struggle with their identity, so making snap judgements is a poor way of understanding others (Student 64).*

*This module made me reflect deeply on how difficult it is to separate others’ perceptions of me from my own identity, and how assumptions can have a huge (often harmful) impact on people (Student 69).*

Two key insights were that others’ perceptions influence how people view themselves, and others’ perceptions and self-perceptions could be barriers to individual patient care.

*… everyone’s individual stories definitely highlighted the way in which other people’s perceptions and assumptions of us can shape our own self-perception. This acuity is important to understanding the barriers of care for each individual patient (Student 71).*

*It made me want to be really conscious of the things I say to people about themselves because I never want to have a negative impact on how someone feels or their identity (Student 66).*

Students perceived that the workshop focused mostly on interpersonal biases rather than structural inequities (e.g. systemic racism, social privilege, institutional inequities).

*This module was much more focused on how to listen and not make assumptions from a stand-up lens rather than examples of structural inequities (Student 73).*

### Space for safe, brave, fun discussions

Many students thought the workshop created the space for safe, brave, fun conversations about difficult topics.

*I liked that this module made us get uncomfortable in a safe space and reflect on questions about perception and assumptions. It was also really fun to be a part of! I love Mona’s [author MA] energy*:*) (Student 69).*

*I really liked how this module focused on thinking about ourselves and taking our character seriously while also framing it through a mode of expression that doesn’t take the subject matter too seriously. This allowed me to think about myself critically while also not placing an extreme amount of pressure on what I found about myself. It also lowered the stakes for what it might mean to encounter others, meaning that it was a good place to practice (Student 64).*

*I surprised myself by how much I enjoyed this! The facilitators made it clear that we were under no pressure to share, and that in turn made me feel more comfortable speaking than I ordinarily would in this sort of setting (Student 73).*

*I was surprised by how much I enjoyed this session. It helped me reflect and learn more about my classmate’s viewpoints on how perceptions affect our actions (Student 70).*

Students suggested ways to increase comfort speaking up.

*I would have probably shared my story if we had gone into smaller breakout rooms, the group still felt a little too big for me to feel comfortable (Student 66).*

*I think that this module was good, but it was stressful for me as someone who is usually uncomfortable with sharing myself in front of others to see my classmates so successfully talk about themselves. This is not an inherently bad thing, but it made me feel a bit inadequate in my self-reflection (Student 64).*

*I wish it could have been held in-person, but that’s not possible right now. I think it would have been a lot more comfortable to speak up in an in-person session (Student 70).*

### Value of standup and the non-medical context

Students perceived standup principles and practices to be valuable for self-understanding, perspective, and effective communication.

*I liked that it challenged me to do something new and also to rethink what it means to do stand-up comedy. I realize now that so much of being a comedian is actually about understanding yourself and leaning into your own life experiences (Student 71).*

*It’s made stand up comedy a little less intimidating, while at the same time increasing my awe for stand up comedians’ storytelling abilities and resilience. I’ve realized that their work is indeed analogous to being a doctor, and I think it is good practice for increasing self-understanding (Student 71).*

*Comedy brings a different perspective on a very important topic (Student 75).*

*It made me more interested in stand-up as a method to improve communication (Student 73).*

The non-medical context for the exercise was helpful for lasting impact.

*I think the more different ways we try to think about our differences in our perspectives aside from medicine, the more likely we are to internalize that lesson and in turn bring it back into medicine with us (Student 75).*

## Discussion

This standup comedy workshop embedded in a health disparities course, engaged first-year medical students in self-reflection and discussions about identity and interpersonal biases, and encouraged them to strive for more accurate, authentic interactions with patients. It is inherently stressful to share self-identity and discuss interpersonal biases honestly in group settings, especially among new classmates. However, all respondents reported that the learning environment was safe, and students engaged in very personal brave discussions around identity and bias [25], building on the key qualities of the successful standup: authenticity, vulnerability, and the personal lived experience.

While the University of Chicago Health Disparities course and other equity courses have had small group discussions with self-reflection [19, 26], our workshop is the first we are aware of that used standup comedy principles and exercises. Why was our workshop well-rated and how scalable and replicable is it? Standup comedy teaches how to recognize the bidirectional perceptions and misperceptions between comic and audience, and the power of saying “No” to the absurdity and social injustice of biases, stereotyping, and racism [8, 20]. The clinician, like the comic, has power over the room and can shape the communication and relationship with the patient to be based on authenticity, trust, and honest discussion. Our workshop enabled introspective reflection on personal identity and misperceptions of others, sharing of these stories and insights with peers, and discussion about stereotypes and biases. In an ethnographic study of Midwestern standup comedians and audiences in America navigating race, DeCamp identified how successful comics would share honest, revelatory personal experiences as a prelude to thought-provoking jokes about racism that challenge stereotypes [27]. No one can deny how you perceive yourself, and the audience getting to know you is the setup for dialogue around racism. The key standup principle in our workshop is honest dialogue based on personal experience as a springboard to discuss difficult issues such as racism and cultural identity [28], not inauthentic performance strategies or rhetorical devices that some comedians use to excuse racist humor [29, 30].

The issues the standup comedian faces interacting with an audience of strangers parallels the clinician’s challenge caring for new diverse patients, and the comedy context is novel, fun, and de-pressurizes the situation. We specifically chose exercises that would not require extensive standup, improv, or comedy training. The more important instructor training and experience were how to create safe, brave space and facilitate difficult conversations around complex, emotionally charged topics [5, 25]. This standup workshop also benefitted from the wider Health Disparities course which had previously introduced the concepts of interpersonal biases, structural inequities, systemic racism, social privilege, civil discourse, and safe space [19].

We started with fun warm up exercises before the personal monologue. Both instructors gave example personal monologues that embodied the standup qualities of authenticity, vulnerability, and personal lived experience. One of the instructors was a woman from an ethnic and religious minority group who was not a health care professional, with rich international lived experience, a very upbeat supportive style, and streetwise legitimacy interacting with diverse standup audiences. Both instructors have extensive teaching experience discussing diversity and bias issues, sharing personal experiences, and facilitating challenging conversations, but these skills do not require standup comedy training.

One hundred percent of the respondents agreed or strongly agreed that the learning environment was safe. Thirty-six percent of respondents reported stress. Some stress could have been related to public speaking and self-disclosure. However, we made clear students did not have to participate in any part of the workshop they did not feel comfortable, they should disclose only what they felt comfortable sharing, and they could contribute in many ways including commenting upon what was openly discussed. In addition, a videoconference safe room staffed by the Health Disparities course director was available for any student who wanted to leave the standup session to discuss or address any personal issue, feeling, or reaction. No students accessed the safe room. The level of stress might be a good thing engaging students in difficult conversation around bias, racism, identity, and privilege, the constructive tension for growth advocated by Reverend Martin Luther King, Jr. [31] Importantly first-year medical students in their second month of medical school were ready and able to benefit from this training.

Our study is an exploratory pilot with small sample size. In addition, our survey was brief and limited by a 42.5% survey response rate. It is possible that the nonrespondents would have rated the workshop less favorably and reported that the learning environment was not safe. However, the surveys did not have personally identifiable information and students did not have to answer any question, lessening the chance of major selection bias among nonrespondents. Given how positive the workshop ratings were, a very high percentage of nonrespondents would have had to answer negatively to change the overall results of the evaluation.

In addition, few respondents self-identified as African American or Latino despite 28% of the overall medical school class being of those identities; 29% of respondents did not answer the demographic question. Thus, we cannot be certain whether the workshop experience differed by race or ethnicity. Future studies should be designed to understand in more detail how different students, especially those from minoritized groups, perceived the course [32]. Those students who responded did openly express what they liked about the workshop and what could be improved, and we did not sense any major negative reaction to the workshop. We do believe our pilot demonstrates promise for using standup principles in health equity teaching. In addition, a strength of our study is that randomly chosen students took our standup workshop, not a subset of volunteers which would likely raise the ratings.

Our standup workshop led to significant discussions about interpersonal biases but not about systemic inequities. Time limited the number of discussion questions we could ask. For example, if we had more time, we could add a question specifically designed to spur dialogue around systemic inequities (e.g. – “How do your personal stories and reflections around identity and interpersonal biases raise questions around how systemic inequities impact health outcomes for our patients?”). Some art forms such as Theater of the Oppressed are specifically designed to spur discussion around systemic inequities [33, 34]. We are trying to determine the best ways to integrate standup comedy, improvisational comedy [20], graphic medicine [35], and Theater of the Oppressed in our training about advancing health equity [21]. Future work should also test scalability and replication, effects of mode of workshop delivery such as Zoom versus in-person, class size, instructor qualifications, training, and competencies, and how best to obtain institutional buy-in.

Further workshops could teach more advanced standup skills important for care of diverse patients. For example, good standup comedians “work the room” to engage the audience in spontaneous dialogue and bond with them. Skilled standup comedians also are effective at using storytelling as a way to communicate and make complex ideas understandable and relatable. Standup comedians also are taught to have an attitude about their topic, classically “What is stupid, weird, scary, or hard about Topic X?“ [7] Those attitudes exaggerate the absurd and unjust, and provide an opening to discussing health disparities, biases, and discrimination [28, 36]. Standup comedians continuously learn and revise rather than being locked in set perceptions. Seemingly spontaneous comedy acts are actually built upon countless trial runs of jokes at open mics in clubs and bars. These standup principles, including pointing out the absurdity of discrimination and saying “No” to injustice [36], complement the improv principle of “Yes, and…” [20], truly listening to the patient and working with the patient starting with where they are at.

## Conclusions

Our standup comedy pilot shows promise for engaging students in meaningful discussions about perceptions and interpersonal biases rooted in their own personal experiences and those of their classmates. While we cite conceptual models and theoretical frameworks [11, 13, 15], our sense as standup comedians and educators is that the power of our workshop comes from the heart [6, 20]. In today’s contentious times, breaking down walls between people will require this high level of self-insight, honesty, and emotional connection to bond with mutual understanding as humans, a key requirement for establishing effective clinician-patient communication and relationships across diverse populations.

## Supplementary Information

**Additional file 1: Appendix.** Post-Session Evaluation Survey

## Data Availability

The datasets generated during and analysed during the current study are not publicly available nor available upon request due to privacy restrictions.
