# Supplementary material for: Standup comedy principles and the personal monologue to explore interpersonal bias: experiential learning in a health disparities course
Source: BMC Med Educ. 2022 Feb 5;22:80. doi: 10.1186/s12909-022-03139-7 (PMC8817666; doi:10.1186/s12909-022-03139-7)
Supplement: Supplementary file 1 — Additional file 1: Appendix. Post-Session Evaluation Survey [file 12909_2022_3139_MOESM1_ESM.docx]

**Appendix. Post-Session Evaluation Survey**

Please check one of the responses for each question:

|  | Strongly Disagree | Disagree | Neutral | Agree | Strongly Agree |
| --- | --- | --- | --- | --- | --- |
| 1. This module helped me become a better listener. | **€** | **€** | **€** | **€** | **€** |
| 1. This module helped me become more observant. | **€** | **€** | **€** | **€** | **€** |
| 1. The learning environment in this module was safe. | **€** | **€** | **€** | **€** | **€** |
| 1. This module helped me bond with my classmates. | **€** | **€** | **€** | **€** | **€** |
| 1. We had meaningful discussions about systemic inequities. | **€** | **€** | **€** | **€** | **€** |
| 1. I felt good about myself in this module. | **€** | **€** | **€** | **€** | **€** |
| 1. The instructors created an atmosphere in which I could take risks. | **€** | **€** | **€** | **€** | **€** |
| 1. This training could help me take better care of patients with lived experiences different than my own. | **€** | **€** | **€** | **€** | **€** |
| 1. I felt stressed during this module. | **€** | **€** | **€** | **€** | **€** |
| 1. I would recommend this module to others. | **€** | **€** | **€** | **€** | **€** |

1. What did you like about this module?
2. What could be improved about this module?
3. What effect did this module have on you?
4. What was your experience with the module discussions about structural inequities (e.g. systemic racism, social privilege, institutional inequities)?
5. Overall, how would you rate this module? (Please check one box)

| Poor | Fair | Good | Very Good | Excellent |
| --- | --- | --- | --- | --- |
| € | **€** | **€** | **€** | **€** |

1. Please indicate your gender
2. Please indicate your race/ethnicity
3. Please describe any prior experience you have in the arts (e.g. – improv, standup, storytelling, graphics, theater, music, etc.).
4. Other comments:

Thank you for completing the survey.
